# Structural and functional brain changes in perimenopausal women who are susceptible to migraine: a study protocol of multi-modal MRI trial

**DOI:** 10.1186/s12880-018-0272-6

**Published:** 2018-09-06

**Authors:** Bo Hu, Xu Wang, Jie-bing He, Yu-jie Dai, Jin Zhang, Ying Yu, Qian Sun, Lin-FengYan, Yu-Chuan Hu, Hai-Yan Nan, Yang Yang, Alan D. Kaye, Guang-Bin Cui, Wen Wang

**Affiliations:** 1 Department of Radiology & Functional and Molecular Imaging Key Lab of Shaanxi Province, Tangdu Hospital, Fourth Military Medical University (Air Force Medical University), 569 Xinsi Road, Xi’an, 710038 Shaanxi China; 2 Student Brigade, Fourth Military Medical University (Air Force Medical University), 169 West Changle Road, Xi’an, 710032 Shaanxi Province China; 3 Department of Clinical Nutrition, Xijing Hospital, The Fourth Military Medical University, Xi’an, China; 4 Departments of Anesthesiology and Pharmacology, Louisiana State University School of Medicine, New Orleans, Louisiana USA

**Keywords:** Perimenopausal, Migraine, Multi-modal, Voxel-based morphology (VBM), Functional connectivity (FC), Diffusional kurtosis imaging (DKI), Artery spin labeling (ASL)

## Abstract

**Background:**

As a common clinical symptom that often bothers midlife females, migraine is closely associated with perimenopause. Previous studies suggest that one of the most prominent triggers is the sudden decline of estrogen during perimenopausal period. Hormone replacement therapy (HRT) is widely used to prevent this suffering in perimenopausal women, but effective diagnostic system is lacked for quantifying the severity of the diseaase. To avoid the abuse and overuse of HRT, we propose to conduct a diagnostic trial using multimodal MRI techniques to quantify the severity of these perimenopausal migraineurs who are susceptible to the decline of estrogen.

**Methods:**

Perimenopausal women suffering from migraine will be recruited from the pain clinic of our hospital. Perimenopausal women not suffering from any kind of headache will be recruited from the local community. Clinical assessment and multi-modal MR imaging examination will be conducted. A follow up will be conducted once half year within 3 years. Pain behavior, neuropsychology scores, fMRI analysis combined with suitable statistical software will be used to reveal the potential association between these above traits and the susceptibility of migraine.

**Discussion:**

Multi-modal imaging features of both healthy controls and perimenopausal women who are susceptible to estrogen decline will be acquired. Imaging features will include volumetric characteristics, white matter integrity, functional characteristics, topological properties, and perfusion properties. Clinical information, such as basic information, blood estrogen level, information of migraine, and a bunch of neurological scale will also be used for statistic assessment. This clinical trial would help to build an effective screen system for quantifying the severity of illness of those susceptible women during the perimenopausal period.

**Trial registration:**

This study has already been registered at Clinical Trials. gov (ID: NCT02820974). Registration date: September 28th, 2014.

## Background

As a common headache disorder, migraine is characterized by recurrent headache attacks accompanied with symptoms including nausea and vomiting [1]. According to the World Health Organization (WHO), migraine is considered as one of the most disabling and burdensome diseases [2, 3]. In general, adult females experience migraine attacks twice more than males, and although men and women reported similar headache severity and frequency, women reported more migraine related symptoms [4, 5].

This gender difference is mainly caused by the difference in sex hormone. Estrogen quantities are relatively stable in men and undergo a much more gradual decline with age as compared with women [6]. For example, previous studies revealed that migraine is closely linked to the menopausal period of women, and one of the most prominent triggers is the sudden decline of estrogen [7–9]. Estrogen can bind to its receptors of nucleus or cytoplasm in the brain, and then exert its function of pain transmission by gene transcription and protein synthesis [10]. However, this procedure would be disturbed by the sudden drop of estrogen in perimenopausal period, which would furtherly result in the susceptibility to migraine [6].

Since the decline of estrogen begins in perimenopausal period, giving hormone replacement therapy (HRT) in this period is considered as an effective method to treat migraine [11, 12]. To avoid the abuse and overuse of HRT which could cause some severe unpleasant complications, those women should be given the lowest effective dose of medication [13, 14]. However, headache is a subjective feeling, and effective diagnostic system is still lacked for quantifying the degree of migraine, which could result in the abuse and overuse of HRT in treating this disease.

In the past two decades, functional magnetic resonance imaging (fMRI) technique has become an indispensable tool in migraine research and has greatly contributed to our understanding of migraine pathophysiology [15–17]. It could show anatomic and functional cerebral transformations associated with the clinical evolution and could be applied to most individuals without adverse effects. Maleki et al. found that female migraineurs have thicker posterior insula and precuneus cortices as compared with both male migraineurs and healthy controls of both sexes [18]. Compared to healthy controls, Kim and colleagues revealed a reduction of gray matter (GM) density in migraineurs, including insula, motor/premotor, prefrontal, cingulate, posterior parietal, and orbitofrontal cortex [19]. Huang et al. found reduced fractional anisotropy (FA) in the right frontal of migraine patients by using a tract-based spatial statistics (TBSS) method, which means the microstructure of white matter (WM) in that region is impaired [20]. Liu et al. found that the resting-state functional connectivity (FC) was disrupted in patients with long-term migraine through the method of graph theory [21]. However, these researches are all focused on both male and female patients at all ages, which contributes little to quantify the degree of migraine in perimenopausal women. According to our knowledge, no previous studies had researched the imaging abnormality of perimenopausal women who are susceptible to the decline of estrogen. Since estrogen plays a pivotal role in pain transmission in our brain [10], we thus raised the current hypothesis that brain structure and function of these women who are susceptible to estrogen drop exhibit unique characteristic changes in multi-modal images.

Consequently, we would like to conduct a prospective clinical trial to investigate the imaging abnormalities in these susceptible perimenopausal women. At the beginning, perimenopausal women around who had migraine will be recruited from the pain clinic of our hospital. Perimenopausal women around who did not have migraine will be recruited as healthy controls (HC) from the local community. Then, these subjects will undergo a series of multi-modal MRI brain scans and neuropsychology scales to assess the mental and physical state of these subjects. Multi-modal MR images of these two groups of subjects will be compared to find significant features as objective biomarkers.

## Methods

**Fig. 1:**
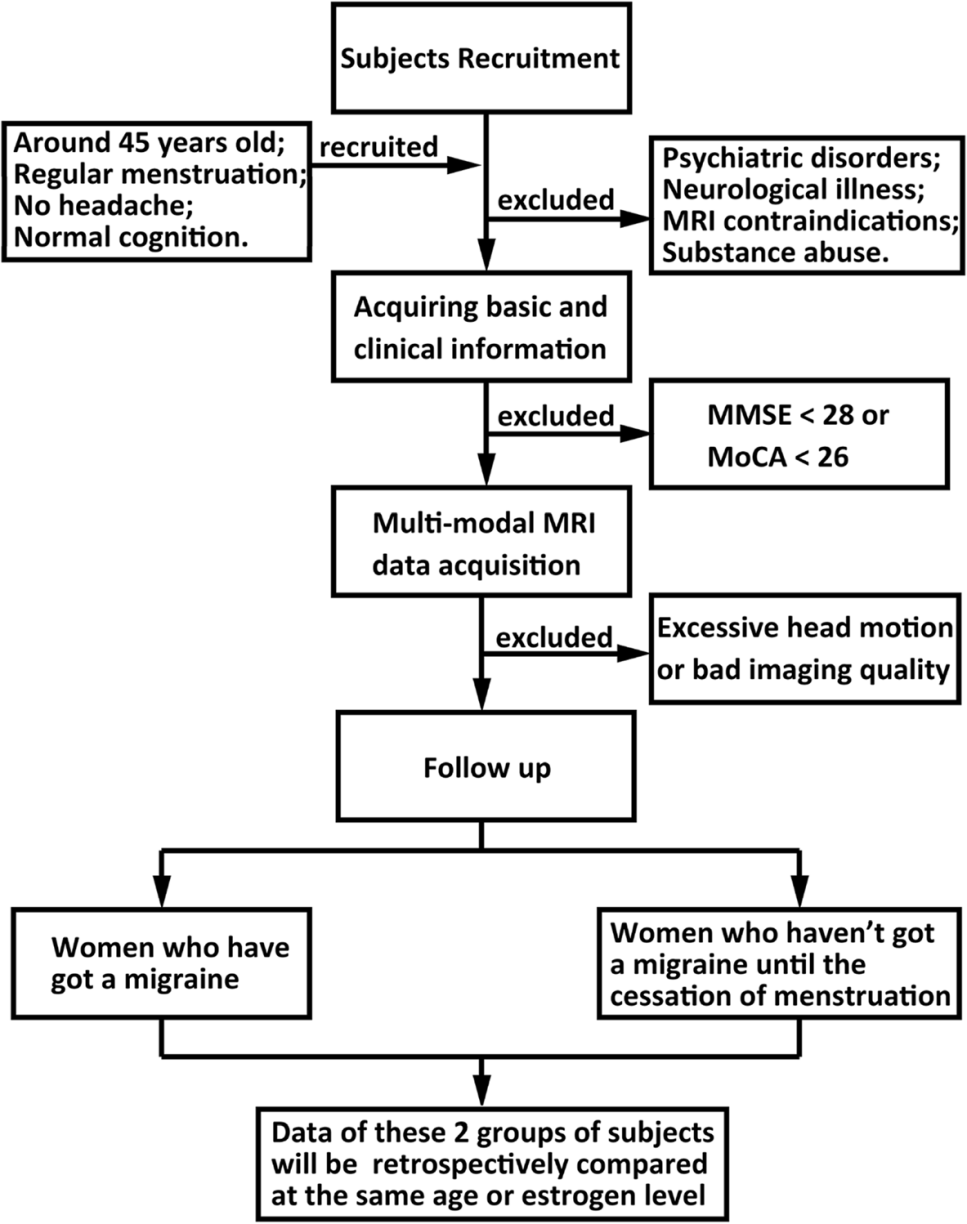
The flowchart of the current trial

The flowchart of the current trial is described in Fig. 1.

### Subject

All subjects will be provided informed, written consents after the study procedure has been fully explained in detail. Females who match the following criteria would be included in the migraine group: (1) age ranges from 40 to 55 years old; (2) irregular menstrual cycle; (3) suffered from migraine; (4) normal neurological examination. Females who match the following criteria would be included in the HC group: (1) age ranges from 40 to 55 years old; (2) irregular menstrual cycle; (3) not suffered from any kinds of headache; (4) normal neurological examination. Exclusion criteria will be as follows: (1) severe psychiatric disorders, such as dementia, episode, epilepsy, major depression; (2) significant neurological illness, such as significant head trauma, tumor, meningitis or central nervous system (CNS) inflammatory lesions and vascular complications clues; (3) any MRI contraindications; (4) alcohol, nicotine or drug abuse.

Sample size have been estimated by referring to other studies [22, 23]. A sample size of 40 patients per group will be suitable for a power of 0.8 and an alpha significance level of 0.05 (two-side). Considering the drop-out ratio (25%) and unqualified images of included subjects, the total sample size is 55 in each group.

### Clinical assessments

Demographic and clinical features of all subjects will be collected and recorded, such as age, education level, body mass index (BMI), occupation and menstrual cycle. All participants will receive the following battery of neuropsychological tests: Montreal Cognitive Assessment (MoCA) and Mini-mental State Examination (MMSE) will be used to assess the mental state of subjects. The Short Form health survey (SF-36) will be applied to evaluate the quality of life, and Self-rating Depression Scale (SDS) and Self-rating Anxiety Scale (SAS) will be performed to measure the severity of self-reported depression and anxiety. Migraine will be diagnosed according to the International Headache Society (IHS) criteria of the International Classification of Headache Disorders (ICHD-3 beta version) [1]. The following data will be collected from migraine participants: (1) type of migraine (migraine with aura, migraine without aura, etc.), (2) frequency of migraine attacks, (3) duration of migraine attacks, (4) headache location (unilateral or bilateral), (5) Migraine Disability Assessment Scale (MIDAS) score (the score is divided into 4 grades, higher grade means more severer disability). Blood samples were drawn at the screening centers and processed within 2 h according to a standardized protocol to assess the hormone level [24].

### Follow up

A follow up will be conducted within three years after individual’s first visit. Individuals will come back to the medical center once half year for countercheck, and all the protocols and evaluations will be the same as that they get at the first time. In addition, medication type and dose will also be recorded.

### Multi-modal MRI examination

Multi-modal imaging examination will be performed with a 3.0-T MRI system (MR750; GE Healthcare, Milwaukee, WI, USA) using an 8-channel head coil array with foam padding to restrict head motion. Multi-modal scans including high-resolution three-dimensional brain volume T1-weighted imaging (T1WI), diffusion kurtosis imaging (DKI), resting-state blood oxygenation level-dependent (RS-BOLD) imaging and three-dimensional pseudo-continuous arterial spin labeling (3D-pCASL) imaging will be conducted to detect microstructural and functional properties. All the sequences we mentioned will use the isotropic spatial resolution.

#### High-resolution T1WI

Sagittal three-dimensional brain volume (3D-BRAVO) sequence will be used for the acquisition of high-resolution T1-weighted images, according to the following parameters: echo time (TE) = 3.2 ms, repetition time (TR) = 8.2 ms, TI = 450 ms, flip angle (FA) = 12°, field of view (FOV) =256 × 256 mm^2^, matrix = 256 × 192, slice thickness = 1.0 mm, slice number = 166. The whole procedure will cost 4 min 43 s.

#### DKI

DKI datasets will be collected using a douple-spin-echo diffusion tensor echo planar imaging (SE-SS-DT-EPI) sequence with a clinically oriented protocol. The acquisition parameters are as follows: six-b-values of 0,500, 1000,1500, 2000, and 2500 s/mm^2^, 10 b_0_ images, 30 diffusion gradient directions, TR = 6500, TE = 108 ms, FOV =256 × 256 mm^2^, matrix = 128 × 128, slice thickness = 3 mm, FA = 90°, slice number = 50, voxel size = 2 mm × 2 mm × 5 mm. The acquisition time for this protocol will be 6 min.

#### RS-bold

RS-BOLD images will be collected using a gradient-recalled echo-planar imaging (GRE-EPI) sequence with the following parameters: TR = 2000 ms, TE = 40 ms, FA = 90°, slice number = 36, inter slice gaps = 0 mm, FOV = 240 × 240 mm^2^, matrix = 64 × 64, slice thickness = 3 mm, in-plane spatial resolution = 3 × 3 mm^2^. Participants will be reminded to lie quietly in the scanner with their eyes closed and thinking nothing during data acquisition. The whole procedure will cost 6 min 10 s.

#### 3D-pCASL

Resting-state perfusion imaging was performed using pCASL sequence with a 3D fast spin-echo acquisition and background suppression: TR = 5046, 11.1 ms, FOV = 128 × 128 mm2, matrix = 128 × 128 mm2, slice thickness = 3 mm, FA = 111°, number of excitation = 3, no gap; 40 axial slices; number of excitation = 3. The whole procedure will cost 4 min 53 s.

### Imaging data processing

#### High-resolution T1WI data

All T1 imaging data will be processed and examined for voxel-based morphological (VBM) analyses using the VBM8 toolbox (http://dbm.neuro.uni-jena.de/vbm/) in Statistical Parametric Mapping (SPM) (SPM8, Wellcome Department of Imaging Neuroscience Group, London, UK; http://www.fil.ion.ucl.ac.uk/spm) running in MATLAB 2014a platform. We will perform an optimized VBM protocol in which a brain-tissue-only template will be used other than a whole-brain template. Brain Extraction Tool (integrated in MRIcro; http://www.mricro.com) was used to remove non-brain region images [25]. After removal of nonbrain regions, images will be segmented into GM, white matter (WM), and cerebrospinal fluid (CSF) by using the Diffeomorphic Anatomical Registration Through Exponentiated Lie algebra (DARTEL) algorithm [26]. Afterward, the normalized images would be averaged by using SPM mean function. To create the final template, all average images would be smoothed using an isotropic Gaussian kernel with a full width at half maximum (FWHM) of 8 mm.

#### DKI data

All DKI images will be processed using a combination of FMRIB’s Software Library (FSL) [27] and in-house image processing tools developed in MATLAB. The diffusion dataset will be modulated to get potential 3D head motion and eddy current distortion using FSL eddy correct. The toolbox implement in MTALAB will be applied to deal with diffusional kurtosis tensors. Region of interest (ROI) of headache will be drawn by hand. DKI parameters of migraine pain-ROIs, such as mean kurtosis (MK), mean diffusivity (MD) and fractional anisotropy (FA), will be measured. All feature maps will be spatially normalized to the standard MNI space by using the transformation fields derived from tissue segmentation of structural images and resampled to 3 mm isotropic voxels.

#### BOLD data

RS-BOLD data will be preprocessed in the toolbox of MATLAB (Data Processing Assistant for Resting-State fMRI, DPARSF; http://www.restfmri.net/forum/DPARSF). SPM8, RS-fMRI data analysis toolkit (REST1.6; http://www.restfmri.net) and graph-theoretical network analysis (GRATNA; https://www.nitrc.org/projects/gretna) will also be selected to deal with the images. The first 10 time points will be discarded to ensure stable magnetization and allow the participants to adapt to the EPI scanning environment. After that, slice timing and head motion will be conducted, and scans with head motion of translation > 3.0 mm or rotation > 3° will be excluded. The functional images will then be spatially normalized to Montreal Neurological Institute (MNI) space using the transformation fields derived from tissue segmentation of individual structural images and resampled to 3 × 3 × 3 mm^3^. The resulting images will be smoothed with 8 mm FWHM isotropic Gaussian kernel. Linear trends will be removed from the image time series, and data will be band-pass filtered at 0.01–0.08 Hz. Finally, nuisance signals such as 24-parameter head motion profiles, white matter, and cerebrospinal fluid signals will be regressed out from each voxel’s time series to exclude non-neuronal sources [28].

Amplitude of low frequency fluctuation (ALFF), regional homogeneity (ReHo) and functional connectivity (FC) values will be estimated in the DPARSFA software that is developed to extract abnormal regions between two groups. Small-world properties and network efficiency will be calculated in the GRETNA software to compare the topological characteristics between two groups.

#### 3D-pCASL data

For the ASL fMRI data, corresponding CBF images were obtained using an automated image postprocessing tool embedded in the GE healthcare MR-750 system. Subsequently, the CBF images will be spatially normalized to the standard MNI space by using the transformation fields derived from tissue segmentation of structural images and resampled to 3 mm isotropic voxels. The resulting images will be transformed to z scores using Fisher’s transformation approach and then will be smoothed with 8 mm FWHM isotropic Gaussian kernel.

### Statistical analysis

Statistical Package for the Social Sciences (SPSS) 18.0 (SPSS, Chicago, IL, USA) will be used to analyze the socio-demographic and clinical data. SPM8 and REST 1.8 will be used to analyze the imaging data. All statistical tests were conducted with a significance level of 5%.

#### Socio-demographic and clinical data

We will use 2-sample Student-t test for discrete quantitative data (age, body mass index, educational level, migraine history, frequency of migraine attacks, duration of migraine attacks, SAS score, SDS score, MMSE score, MoCA score and MIDAS scores), and the chi-square (χ2) test for nominal qualitative data (gender, type and location of migraine).

#### Structural parameters

Both voxel-based and ROI-based 2-sample Student-t test will be applied to compare global gray matter (GMV) MK, MD and FA between susceptible group and healthy control group with age, sex, education and BMI as covariance. Moreover, non-parametric Spearman correlation will be performed to delineate a possible relationship between local morphologic alterations and clinical data as well as diffusional alterations and clinical data. Significance threshold, which is corrected for comparison using Family Wise Error (FWE) method, will be set at *P* < 0.05. For each design matrix, significant effects both in the negative and positive directions are considered.

#### Functional and perfusion parameters

Both voxel-based and ROI-based 2-sample Student-t test will be applied to compare global ReHo, ALFF/fALFF, CBF between susceptible group and healthy control group with age, sex, education and BMI as covariance. The reference time series for headache-related seed regions will be acquired by averaging the time series for all voxels within them. Correlations of this time-series will be calculated to measure FCs among these regions in each participant. To evaluate group differences in the resting-state topological properties, nonparametric permutation tests were applied [29]. Initially, we will calculate the between-group differences of the mean values for each topological property. Next, an empirical distribution of the difference will be obtained by randomly reallocating every property value into two groups and recomputing the mean differences between the two randomized groups (5,000 permutations) to test the null hypothesis that the observed group differences only occurred by chance. The 95th percentile points of each distribution will be used as critical values for a two-tailed test to determine whether the observed group differences could occur by chance. Non-parametric Spearman correlation will also be performed to delineate a possible relationship between functional alterations and clinical data as well as perfusion alterations and clinical data. Significance threshold will be set at *P* < 0.05 (FWE corrected).

### Correlation analysis

Linear regression analysis will be performed across the migraine groups to assess relationships between imaging properties and cognitive testing scores and clinical information. When the most sensitive parameters are found, a receiver operating characteristic (ROC) curve will be plotted to testify the sensitivity and specificity of the certain parameter for quantifying the degree of migraine and screening the accompanied cognitive decline.

### Outcomes measure

#### Primary outcomes measure

White matter integrity (e.g. MD, MK and FA changes),

#### Secondary outcomes measure

Headache level (1–10, VAS), volumetric characteristics (e.g. GMV atrophy), functional characteristics (e.g. ReHo, ALFF/fALFF and FC and abnormalities), topological properties (e.g. small-world properties and network efficiency) and perfusion properties (e.g. CBF changes).

## Discussion

Multi-modal imaging features of perimenopausal women who are susceptible to estrogen decline will be acquired. Imaging sequences will include 3D-BRAVO, DKI, RS-BOLD and 3D-pCASL. Imaging features for assessment will include volumetric characteristics, white matter integrity, functional characteristics, topological properties and perfusion properties. Clinical information, such as information of migraine, headache level, MIDAS score, SAS, SDS, MMSE, MoCA and SF-36 will also be acquired for assessment.

The structural imaging could reflect the long-term impact of migraine on the brain structure, including GM volume and WM integrity. VBM has been pervasively used to investigate structural changes in migraine in the last decade. Some of them observed significant GM abnormalities in several brain regions of migraine patients [30–32], including right superior temporal gyrus, right inferior frontal gyrus and left precentral gyrus etc., but the result in other studies were not significant [33–35]. Two recent meta-analysis considered that migraine could cause GMV changes, and these changes were related to gender [36]. The integrity of WM is usually measured by calculating several parameters of its diffusional ability, such as MD, MK and FA. Several previous studies had compared these parameters between migraine patients and healthy controls, and they found significant increases or decreases in brain regions like thalami, corpus callosum and internal capsule [37–40]. Previous studies had also investigated the difference of WM integrity between migraine patients with and without aura and between ictal and interictal period [37, 41]. In addition, imaging researches about WM integrity in children or adolescent who have migraine had also been conducted [42, 43]. In the current study, we hope that the long-term impact of the susceptibility of estrogen drop on brain structure could also be reflected in structural imaging.

The functional imaging could reflect the impact of migraine on the brain activity, including neural activity and perfusion condition. RS-BOLD signal had attracted considerable attention in migraine patient to examine potential alterations of brain induced by recurrent migraine attacks. In migraine patients, previous studies have detected various alterations regarding functional connectivity in pain related regions including the periaqueductal gray area (an important region involved in nociceptive processing) [44], as well as insular which is stronger functionally coupled to the dorsal caudal pons and periaqueductal gray area than healthy controls [45]. Other recent studies have reported changed ReHo, ALFF/fALFF or FC in the executive control network [46, 47], and the default mode network [35]. In addition, the functional topologic properties of migraineurs were also observed in precentral gyrus, anterior cingulate gyrus, thalamus and etc. [21, 48]. Previous researches had identified CBF abnormalities as an imaging biomarker in migraineurs, but these CBF studies were all based on single-photon emission computed tomography (SPECT), which is invasive and difficult to generalize in clinical trial [49, 50]. Recently, ASL is an emerging MRI sequence that can be easily performed without contrast injection or any side effects. Some recent studies stressed the significant abnormalities in somatosensory cortex or other brain regions of migraineurs [51, 52], while one study declared that they didn’t see and significant changes [53]. We hope that the impact of the susceptibility of estrogen drop on brain activity could also be reflected in functional imaging.

### Trial status

This is the first version of the study protocol, and the recruitment had begun in July 2016, and it will be completed in around 2020.

## Abbreviations

3D-pCASL: three-dimensional pseudo-Continuous Arterial Spin Labeling
ALFF: Amplitude of Low Frequency Fluctuation
DKI: Diffusion Kurtosis Imaging
FA: Fractional Anisotropy
FA: Fractional Anisotropy
FC: Functional Connectivity
fMRI: functional Magnetic Resonance Imaging
GM: Gray Matter
HRT: Hormone Replacement Therapy
MD: Mean Diffusivity
MK: Mean Kurtosis
MMSE: Mini-Mental State Examination
MoCA: Montreal Cognitive Assessment
ReHo: Regional Homogeneity
ROI: Region Of Interest
RS-BOLD: Resting-State Blood Oxygenation Level-Dependent
SAS: Self-rating Anxiety Scale
SDS: Self-rating Depression Scale
SF-36: Short Form health survey
T1WI: T1-Weighted Imaging
TBSS: Tract-Based Spatial Statistics
WM: White Matter
